# Impact of stress hyperglycemia ratio on mortality in patients with critical acute myocardial infarction: insight from american MIMIC-IV and the chinese CIN-II study

**DOI:** 10.1186/s12933-023-02012-1

**Published:** 2023-10-21

**Authors:** Jin Liu, Yang Zhou, Haozhang Huang, Rui Liu, Yu Kang, Tingting Zhu, Jielan Wu, Yuwei Gao, Yuqi Li, Chenyang Wang, Shiqun Chen, Nianjin Xie, Xueyan Zheng, Ruilin Meng, Yong Liu, Ning Tan, Fei Gao

**Affiliations:** 1 Department of Cardiology, Guangdong Provincial People’s Hospital, Guangdong Cardiovascular Institute, Southern Medical University, Guangzhou, China; 2 Guangdong Provincial Key Laboratory of Coronary Heart Disease Prevention, Guangdong Provincial People’s Hospital, Guangdong Academy of Medical Sciences, Guangzhou, China; 3 School of Mathematics, South China University of Technology, Guangzhou, China; 4 Department of Engineering Science, Institute of Biomedical Engineering, University of Oxford, Oxford, UK; 5 The Second School of Clinical Medicine, Southern Medical University, Guangzhou, China; 6 Jinan university, Zhuhai People’s Hospital, Zhuhai, China; 7 Department of Cardiology, Zhongshan City People’s Hospital, Zhongshan, China; 8 Global Health Research Center, Guangdong Provincial People’s Hospital, Guangdong Academy of Medical Science, Guangzhou, China; 9 Institute of Control and Prevention for Chronic Non-infective Disease, Guangdong Provincial Center for Disease Control and Prevention, Guangzhou, China; 10 Department of Cardiology, Beijing Anzhen Hospital, Capital Medical University, Beijing, China

**Keywords:** Coronary artery disease, Stress hyperglycemia ratio, Diabetes, Mortality

## Abstract

**Background:**

Among patients with acute coronary syndrome and percutaneous coronary intervention, stress hyperglycemia ratio (SHR) is primarily associated with short-term unfavorable outcomes. However, the relationship between SHR and long-term worsen prognosis in acute myocardial infarction (AMI) patients admitted in intensive care unit (ICU) are not fully investigated, especially in those with different ethnicity. This study aimed to clarify the association of SHR with all-cause mortality in critical AMI patients from American and Chinese cohorts.

**Methods:**

Overall 4,337 AMI patients with their first ICU admission from the American Medical Information Mart for Intensive Care (MIMIC)-IV database (n = 2,166) and Chinese multicenter registry cohort Cardiorenal ImprovemeNt II (CIN-II, n = 2,171) were included in this study. The patients were divided into 4 groups based on quantiles of SHR in both two cohorts.

**Results:**

The total mortality was 23.8% (maximum follow-up time: 12.1 years) in American MIMIC-IV and 29.1% (maximum follow-up time: 14.1 years) in Chinese CIN-II. In MIMIC-IV cohort, patients with SHR of quartile 4 had higher risk of 1-year (adjusted hazard radio [aHR] = 1.87; 95% CI: 1.40–2.50) and long-term (aHR = 1.63; 95% CI: 1.27–2.09) all-cause mortality than quartile 2 (as reference). Similar results were observed in CIN-II cohort (1-year mortality: aHR = 1.44; 95%CI: 1.03–2.02; long-term mortality: aHR = 1.32; 95%CI: 1.05–1.66). In both two group, restricted cubic splines indicated a J-shaped correlation between SHR and all-cause mortality. In subgroup analysis, SHR was significantly associated with higher 1-year and long-term all-cause mortality among patients without diabetes in both MIMIC-IV and CIN-II cohort.

**Conclusion:**

Among critical AMI patients, elevated SHR is significantly associated with and 1-year and long-term all-cause mortality, especially in those without diabetes, and the results are consistently in both American and Chinese cohorts.

**Supplementary Information:**

The online version contains supplementary material available at 10.1186/s12933-023-02012-1.

## Introduction

Acute myocardial infarction (AMI) remains a growing threat to public health and a leading cause of morbidity and mortality worldwide [1]. According to a nationwide cohort study, nearly 50% AMI patients admitted into intensive care unit (ICU) in the United States, and the mortality remained in the range of 14–50% in last two decades [2–6].

Stress hyperglycemia has been reported in a transient metabolic response with increasing of glucose level among patients with emergency situation [7–9], and caused the atherosclerotic plaque instability, rupture and exacerbate myocardial ischemia [10, 11]. Compared with admission blood glucose (ABG), stress hyperglycemia ratio (SHR) as a newly indicator of stress hyperglycemia which is divided ABG measurement by HbA1c [12–15].

Previous studies on patients with acute coronary syndrome patients or AMI, including those underwent percutaneous coronary intervention, found that SHR has J-shaped or U-shaped association with short- and long-term mortality [12, 16, 17]. While AMI patients who requiring intensive care may have nearly 2-fold mortality increased than non-critical status [4]. It still lacking evidence of the association of SHR with long-term worsen prognosis among AMI patients in critical status. Furthermore, whether this correlation has heterogeneous due to differences ethnicity or not is unknowing.

Accordingly, the purpose of the current study was to assess the relationship between SHR and 1-year and long-term all-cause mortality in AMI patients following admission to ICU from American MIMIC-IV cohort and Chinese CIN-II cohort.

## Methods

### Study design and population

We employed electronic hospital records (EHR) data obtain from one U.S. public critical care databases as well as proprietary real-world EHR dataset from China. The Medical Information Mart for Intensive Care (MIMIC-IV 2.2 version) database provided information on 315,460 inpatients of Beth Israel Deaconess Medical Center from 2008 to 2019 for the American cohort [18]. Patients meeting the following criteria were included: (1) age over 18 years; (2) combined with AMI; (3) first admitted to ICU. Patients meeting the following criteria were excluded: (1) missing discharge status; (2) insufficient or missing important laboratory results (glucose on admission or glycated hemoglobin [HbA1c]); (3) missing follow-up information. Finally, 2,166 critical AMI patients in were included in American MIMIC-IV.

**Fig. 1:**
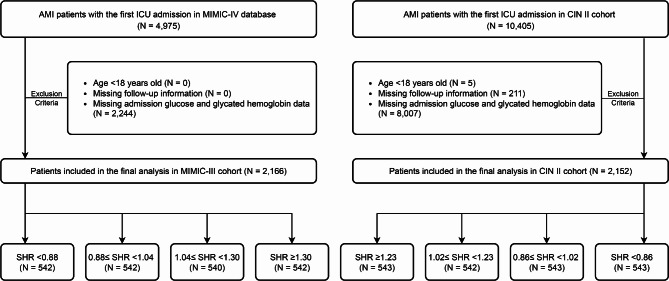
**Flow chart** AMI = acute myocardial infarction, ICU = intensive care unit, SHR = stress hyperglycemia ratio

In the Chinese cohort, we analyzed data in the registry of Cardiorenal ImprovemeNt II (CIN-II, NCT05050877) cohort during 2007 to 2020 in five south Chinese regional central tertiary teaching hospitals, which covered 145,267 patients with coronary catheterization [19]. The same inclusion and exclusion criteria were applied. Finally, 2,171 participants in CIN-II were included. (Fig. 1)

The protocol of CIN-II study was approved by the Guangdong Provincial People’s Hospital Ethics Committee (No.GDREC2019-555 H-2), all participating sites received institutional review board approval from their own ethics committees, and the study was performed according to the declaration of Helsinki.

### Data collection

To access the database, we completed a training course on National Institutes of Health website and an exam of “Protecting Human Research Participants” (certification number: 36,322,632). We extracted variables including demographics, comorbidities, laboratory results, laboratory indices, operations, medication, and discharge status from MIMIC-IV database. The code for data query and extraction was available from the Repository (website: https://github.com/MIT-LCP/mimic-code).

In CIN-II cohort, the in-hospital patient data was collected from the electronic clinical management system, which also contained records of demographic characteristic, clinical variables, and discharge status. The follow-up information was acquired by matching the survival data from Centers for Disease Control and Prevention. Senior cardiologists regularly conducted periodic data verification procedures to ensure data integrity and accuracy.

### Outcomes and definitions

The study outcome was all-cause mortality (maximum follow-up of 12.1 years for MIMIC-IV and maximum follow-up of 14.1 years for CIN-II cohort). SHR was defined as the index calculated by the formula: SHR = (admission glucose) (mmol/L) / (1.59 * HbA1c [%] – 2.59). Chronic kidney disease (CKD) was defined using an estimated glomerular filtration rate ≤ 60 ml/min/1.73 m^2^ by CKD-EPI equation [20]. Anemia was defined as hematocrit below 36% for women and 39% for men [21]. Other comorbidities were identified by ICD-9 or ICD-10 codes of discharge diagnoses in MIMIC-IV database, and by ICD-10 codes in CIN-II cohort.

### Statistical analysis

We divided patients into 4 groups based on quartile of SHR level in MIMIC-IV and CIN-II cohorts, respectively. Continuous data were shown as mean (SD) for data with normally distributed and median (IQR) for data with non-normally distributed, and categorical data were expressed as counts and percentages. ANOVA analysis, the Kruskal-Wallis test for continuous variables, or the chi-square test for categorical data, as applicable, were used to compare the groups. Cumulative hazard of all-cause mortality was estimated by the Kaplan–Meier method.

Univariable and multivariable Cox regression models were performed to assess the relationship between SHR level and long-term all-cause mortality in both two cohorts. Characteristic variables with significant baseline differences or clinical significance and common in the two cohorts were included in final multivariable regression models. Model 1 was unadjusted, model 2 adjusted for age and gender, and model 3 adjusted for age, gender, hypertension, diabetes mellitus, CKD, congestive heart failure (CHF), atrial fibrillation, stroke, anemia, and revascularization (including percutaneous coronary intervention [PCI] and coronary artery bypass grafting [CABG]). In addition, restricted cubic spline (RCS) was used to explore the correlation between SHR and all-cause mortality, with the same adjusted factors as multivariable regression in the RCS model. Hazard ratios (HR) and 95% confidence intervals (CI) are used to present the results. In addition, subgroup analyses were also performed to assess the influence of SHR on all-cause mortality in different subgroups stratified by diabetes (yes or not) and quick sequential organ failure assessment (qSOFA) score (≥ 2 or < 2) (Supplemental Table 1). The statistical analysis was performed by R software (version 4.2.1). A two-tailed P value < 0.05 was considered statistically significant.

## Results

### Baseline characteristics

A total of 1,343 critical AMI patients were enrolled from American MIMIC-IV cohort (mean age [SD]: 67.4 ± 12.9 years; 31.6% female), and 2,171 critical patients with AMI were enrolled in Chinese CIN-II cohort (mean age [SD]: 62.9 ± 12.1 years; 20.6% female). We divided patients into 4 groups based on their SHR level in MIMIC-IV cohort (quartile 1 [n = 542], SHR < 0.88; quartile 2 [n = 542], 0.88 ≤ SHR < 1.04; quartile 3 [n = 540], 1.04 ≤ SHR < 1.30; quartile 4 [n = 542], SHR ≥ 1.30) and CIN-II cohort (quartile 1 [n = 543], SHR < 0.86; quartile 2 [n = 543], 0.86 ≤ SHR < 1.02; quartile 3 [n = 542], 1.02 ≤ SHR < 1.23; quartile 4 [n = 543], SHR ≥ 1.23).

In the MIMIC-IV cohort, 66.3% patients (n = 1,435) had hypertension, 44.1% patients (n = 956) had diabetes, 44.6% patients (n = 966) had CHF and 36.5% patients (n = 790) had CKD, and 37.8% patients (n = 498) received PCI therapy. In addition, 7.0% patients (n = 152) had qSOFA score ≥ 2. In CIN-II cohort, 50.1% patients (n = 1,088) had hypertension, 42.1% patients (n = 915) had diabetes, 42.5% patients (n = 923) had CHF and 32.7% patients (n = 710) had CKD, 89.3% patients (n = 1,938) received PCI therapy, and 20.8% patients (n = 187) had qSOFA score ≥ 2. More details of the baseline information of both cohorts are listed in Table 1.

**Table 1:** Comparison of baseline characteristics between groups stratified by quartile of SHR

Characteristics	Overall	Quartile 1	Quartile 2	Quartile 3	Quartile 4	P value	Overall	Quartile 1	Quartile 2	Quartile 3	Quartile 4	P value
	(**MIMIC-IV)**	< 0.88	0.88–1.04	1.04–1.30	≥ 1.30		**(CIN-II)**	< 0.86	0.86–1.02	1.02–1.23	≥ 1.23	
	N = 2166	N = 542	N = 542	N = 540	N = 542		N = 2171	N = 543	N = 543	N = 542	N = 543	
SHR	1.16 (0.56)	0.75 (0.12)	0.96 (0.05)	1.15 (0.07)	1.77 (0.81)	< 0.001	1.08 (0.34)	0.73 (0.10)	0.94 (0.05)	1.12 (0.06)	1.53 (0.31)	< 0.001
Age, years	69.0 (12.3)	69.2 (12.2)	69.1 (12.5)	68.1 (12.8)	69.6 (11.8)	0.191	62.9 (12.1)	62.7 (11.8)	62.2 (12.0)	62.3 (12.6)	64.2 (12.0)	0.018
Female	665 (30.7)	167 (30.8)	158 (29.2)	154 (28.5)	186 (34.3)	0.161	447 (20.6)	105 (19.3)	101 (18.6)	120 (22.1)	121 (22.3)	0.310
Medical history												
STEMI	604 (27.9)	96 (17.7)	153 (28.2)	166 (30.7)	189 (34.9)	< 0.001	1556 (71.7)	353 (65.0)	412 (75.9)	394 (72.7)	397 (73.1)	< 0.001
NSTEMI	1443 (66.6)	425 (78.4)	364 (67.2)	344 (63.7)	310 (57.2)	< 0.001	362 (16.7)	99 (18.2)	76 (14.0)	88 (16.2)	99 (18.2)	0.189
Hypertension	1435 (66.3)	364 (67.2)	346 (63.8)	372 (68.9)	353 (65.1)	0.308	1088 (50.1)	270 (49.7)	253 (46.6)	277 (51.1)	288 (53.0)	0.187
Congestive heart failure	966 (44.6)	190 (35.1)	212 (39.1)	250 (46.3)	314 (57.9)	< 0.001	923 (42.5)	230 (42.4)	204 (37.6)	230 (42.4)	259 (47.7)	0.010
Diabetes mellitus	956 (44.1)	260 (48.0)	187 (34.5)	211 (39.1)	298 (55.0)	< 0.001	915 (42.1)	256 (47.1)	187 (34.4)	201 (37.1)	271 (49.9)	< 0.001
Chronic kidney disease	790 (36.5)	180 (33.2)	163 (30.1)	186 (34.4)	261 (48.2)	< 0.001	710 (32.7)	169 (31.1)	147 (27.1)	166 (30.6)	228 (42.0)	< 0.001
Atrial fibrillation	753 (34.8)	203 (37.5)	166 (30.6)	171 (31.7)	213 (39.3)	0.005	147 (6.8)	36 (6.6)	31 (5.7)	41 (7.6)	39 (7.2)	0.643
Stroke	365 (16.9)	82 (15.1)	92 (17.0)	83 (15.4)	108 (19.9)	0.130	166 (7.6)	44 (8.1)	39 (7.2)	33 (6.1)	50 (9.2)	0.255
Anemia	1223 (56.5)	297 (54.8)	290 (53.5)	309 (57.2)	327 (60.3)	0.113	885 (42.0)	217 (41.5)	217 (41.1)	213 (40.5)	238 (44.7)	0.502
qSOFA score ≥ 2	152 (7.0)	36 (6.6)	28 (5.2)	43 (8.0)	45 (8.3)	0.166	187 (20.8)	44 (18.8)	44 (20.2)	40 (19.7)	59 (24.1)	0.500
Procedure information												
PCI therapy	295 (13.6)	53 (9.8)	93 (17.2)	81 (15.0)	68 (12.5)	0.003	1938 (89.3)	460 (84.7)	495 (91.2)	496 (91.5)	487 (89.7)	< 0.001
CABG	492 (22.7)	165 (30.4)	136 (25.1)	114 (21.1)	77 (14.2)	< 0.001	22 (1.0)	5 (0.9)	7 (1.3)	5 (0.9)	5 (0.9)	0.908
RRT	63 (2.9)	10 (1.8)	10 (1.8)	18 (3.3)	25 (4.6)	0.017	146 (6.7)	31 (5.7)	29 (5.3)	28 (5.2)	58 (10.7)	< 0.001
Laboratory indexes												
Leukocyte, 10^9/L	10.9 (7.3)	9.5 (7.9)	10.1 (4.2)	11.3 (9.4)	12.7 (6.1)	< 0.001	11.3 (4.3)	10.1 (3.8)	11.1 (3.9)	11.7 (4.4)	12.4 (4.8)	< 0.001
Lymphocyte, 10^9/L	2.0 (5.2)	2.7 (10.0)	1.9 (1.1)	1.8 (2.2)	1.5 (1.0)	0.018	1.6 (0.7)	1.8 (0.7)	1.6 (0.7)	1.5 (0.7)	1.4 (0.7)	< 0.001
Neutrophile, 10^9/L	10.2 (5.5)	8.8 (4.5)	10.0 (5.0)	10.2 (6.0)	11.5 (5.9)	< 0.001	9.0 (4.0)	7.4 (3.3)	8.7 (3.5)	9.5 (4.0)	10.3 (4.4)	< 0.001
Hemoglobin, g/L	122.1 (21.6)	122.8 (20.2)	124.1 (20.9)	122.3 (21.3)	119.0 (23.5)	0.001	130.0 (20.6)	129.6 (19.4)	131.2 (19.7)	130.9 (20.6)	128.5 (22.5)	0.132
LDLC, mmol/L	5.2 (2.4)	5.3 (2.2)	5.2 (2.3)	5.3 (2.1)	5.1 (3.0)	0.894	3.1 (1.0)	3.1 (1.0)	3.1 (1.0)	3.1 (1.1)	3.0 (1.0)	0.295
HDLC, mmol/L	2.5 (0.9)	2.4 (0.8)	2.5 (0.9)	2.4 (0.8)	2.6 (1.0)	0.153	1.0 (0.3)	1.0 (0.3)	1.0 (0.3)	1.0 (0.3)	1.0 (0.3)	0.030
SCr. mg/dL	1.4 (1.4)	1.3 (1.3)	1.4 (1.4)	1.4 (1.2)	1.6 (1.6)	0.000	1.3 (1.1)	1.3 (1.2)	1.2 (1.0)	1.2 (1.0)	1.5 (1.3)	< 0.001
FPG, mmol/L	8.9 (5.1)	6.2 (1.8)	7.2 (2.2)	8.6 (3.0)	13.8 (7.1)	< 0.001	8.5 (3.6)	6.0 (1.7)	7.2 (2.1)	8.7 (2.8)	12.2 (4.1)	< 0.001
HbA1c, %	6.5 (1.6)	6.9 (1.8)	6.3 (1.4)	6.3 (1.6)	6.6 (1.6)	< 0.001	6.6 (1.5)	6.9 (1.6)	6.5 (1.4)	6.5 (1.5)	6.6 (1.5)	< 0.001
Medication												
ACEI/ARB	1265 (58.4)	308 (56.8)	338 (62.4)	319 (59.1)	300 (55.4)	0.101	1383 (71.2)	341 (69.3)	362 (72.0)	358 (72.5)	322 (70.9)	0.704
β-Blocker	2056 (94.9)	526 (97.0)	527 (97.2)	512 (94.8)	491 (90.6)	< 0.001	1582 (81.4)	386 (78.5)	413 (82.1)	400 (81.0)	383 (84.4)	0.128
Statins	2036 (94.0)	515 (95.0)	521 (96.1)	500 (92.6)	500 (92.3)	0.017	1764 (90.8)	441 (89.6)	459 (91.3)	449 (90.9)	415 (91.4)	0.770
Aspirin	2094 (96.7)	534 (98.5)	536 (98.9)	513 (95.0)	511 (94.3)	< 0.001	1805 (92.9)	450 (91.5)	468 (93.0)	468 (94.7)	419 (92.3)	0.227
Diuretic	1661 (76.7)	443 (81.7)	396 (73.1)	408 (75.6)	414 (76.4)	0.007	466 (24.0)	113 (23.0)	104 (20.7)	112 (22.7)	137 (30.2)	0.004
OAD												
Insulin	1719 (79.4)	468 (86.3)	402 (74.2)	400 (74.1)	449 (82.8)	< 0.001	64 (3.3)	20 (4.1)	14 (2.8)	14 (2.8)	16 (3.5)	0.629

Data are means ± SD, median (interquartile range), or n (%)

ACEI/ARB = angiotensin converting enzyme inhibitor or angiotensin receptor blocker, CABG: coronary artery bypass grafting, HbA1c = glycated hemoglobin, HDLC = high density lipoprotein cholesterol, LDLC = low density lipoprotein cholesterol, SCr = Serum Creatinine. qSOFA = quick sequential organ failure assessment, PCI = percutaneous coronary intervention, SHR = stress hyperglycemia ratio, RRT = renal replacement therapy

### Clinical outcomes

**Fig. 2:**
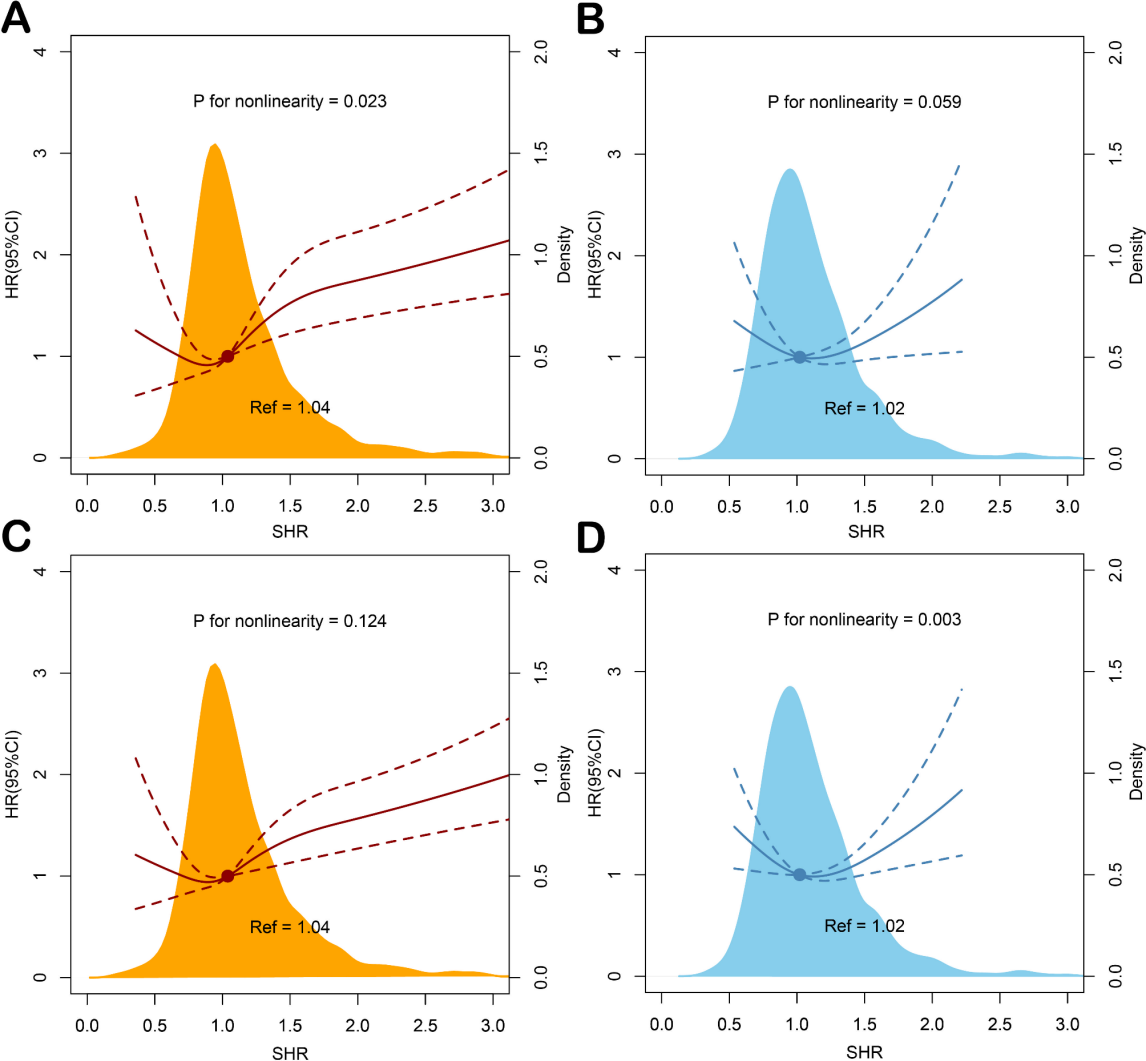
**Association of SHR and all-cause mortality among U.S and Chinese patients with critical AMI** (**A**) SHR and all-cause mortality at 1-year follow-up in U.S MIMIC-IV cohort. (**B**) SHR and all-cause mortality at 1-year follow-up in Chinese CIN-II cohort. (**C**) SHR and all-cause mortality at a maximum follow-up of 12.1 years American MIMIC-IV cohort. (**D**) SHR and all-cause mortality at a maximum follow-up of 14.1 years in Chinese CIN-II cohort. Both cohorts adjusted for age, gender, hypertension, diabetes mellitus, congestive heart failure, chronic kidney disease, atrial fibrillation, stroke, anemia, and revascularization. HRs and 95% CIs are indicated by red lines for MIMIC-IV cohort, and by blue lines for CIN-II cohort. Density plot are presented by orange shadow area for MIMIC-IV cohort, and by light blue shadow area for CIN-II cohort. Ref = reference value

In the American MIMIC-IV cohort, totally 319 patients (23.8%) experienced all-cause mortality, with the highest mortality in quartile 4 (n = 109, 32.4%). Mortality in Chinese CIN-II cohort is similar to American MIMIC-IV cohort: there were 632 cases (29.1%) of all-cause mortality, with the highest mortality in quartile 4 (n = 184, 33.9%). Kaplan–Meier survival analyses results about the two cohorts are presented in Fig. 2.

**Fig. 3:**
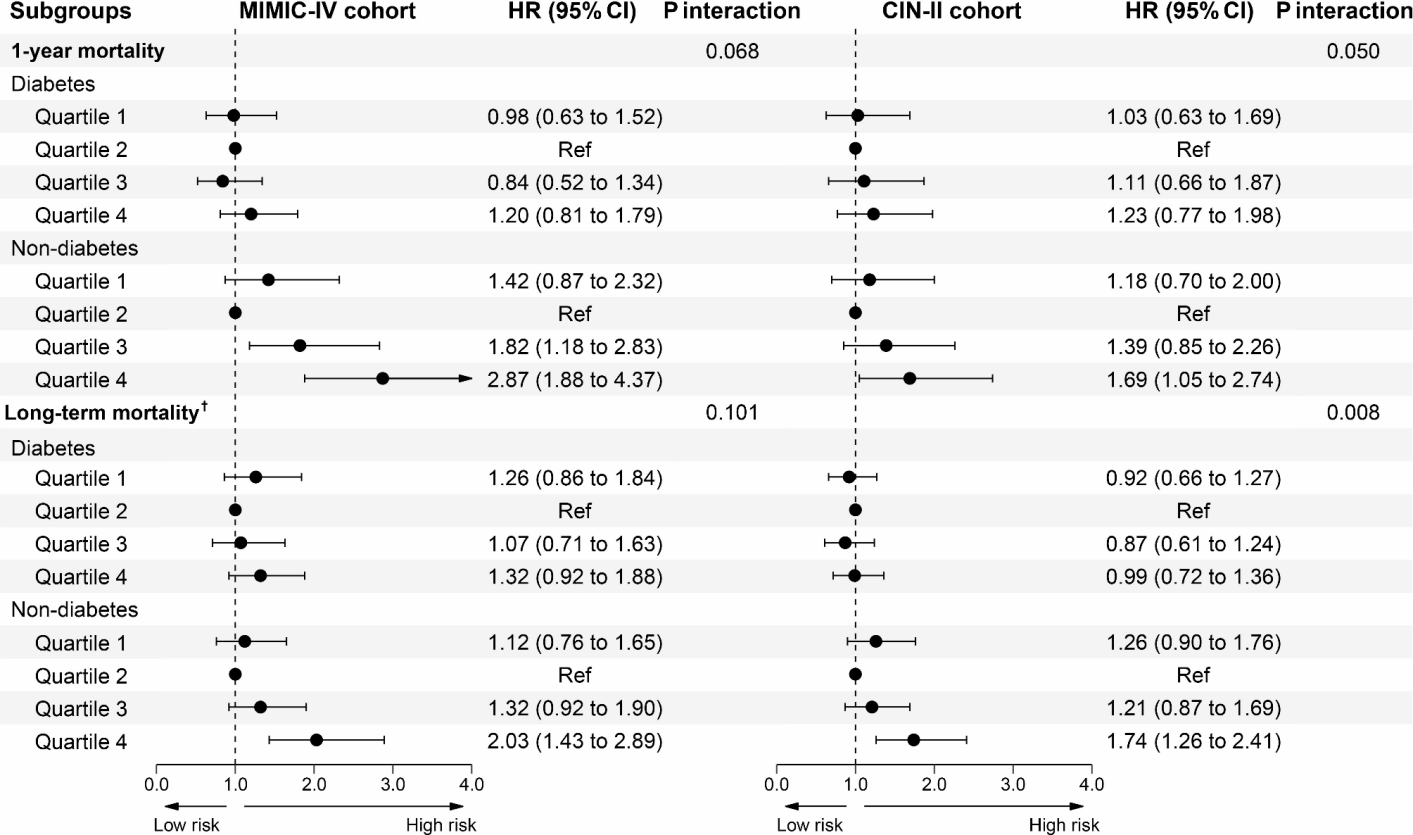
**Forest plot for the associations of SHR and all-cause mortality among patients with or with diabetes mellitus in U.S. and Chinese critical AMI patients** †: Maximum follow-up of 12.1 years for American MIMIC-IV cohort, and 14.1 years for Chinese CIN-II cohort. Both cohorts adjusted for age, gender, hypertension, congestive heart failure, chronic kidney disease, atrial fibrillation, stroke, anemia, and revascularization

Results of the RCS analyses indicated a J-shaped association of the SHR with the all-cause mortality in both two cohorts during follow-up period (P value for nonlinearity < 0.05 for 1-year mortality in MIMIC-IV and for long-term mortality in CIN-II cohort). The cut-off value of SHR to the lowest risk of all-cause mortality on RCS was 1.04 in MIMIC-IV and 1.02 in CIN-II cohort. (Figure. 2)

After adjusted for confounders, in MIMIC-IV cohort, high SHR (as continuous variables) was significantly associated with the risk of 1-year (HR = 1.30 95%CI: 1.19–1.42, P < 0.001) and long-term (HR = 1.30 95%CI: 1.19–1.41, P < 0.001) all-cause mortality. In addition, compared with patients with an SHR of 0.88–1.04 (quartile 2), patients with an SHR ≥ 1.30 (quartile 4) had a higher risk of both 1-year (HR = 1.87 95%CI: 1.40–2.50, P < 0.001) and long-term (HR = 1.63 95%CI: 1.27–2.09, P < 0.001) all-cause mortality. (Table 2)

**Table 2:** Multivariable cox regression analysis for 1-year and long-term all-cause mortality

Groups	MIMIC-IV			CIN-II		
	Events (rate, %)	HR (95%CI)	P value	Events (rate, %)	HR (95%CI)	P value
1-year all-cause mortality						
SHR (continuous)	381 (17.6)	1.30 (1.19–1.42)	< 0.001	298 (13.7)	1.34 (1.01–1.78)	0.043
SHR (categorical)						
Quartile 1	79 (14.6)	1.22 (0.88–1.68)	0.239	71 (13.1)	1.14 (0.80–1.64)	0.459
Quartile 2	70 (12.9)	Ref		55 (10.1)	Ref	
Quartile 3	85 (15.7)	1.30 (0.94–1.78)	0.110	74 (13.7)	1.27 (0.89–1.81)	0.191
Quartile 4	147 (27.1)	1.87 (1.40–2.50)	< 0.001	98 (18.0)	1.44 (1.03–2.02)	0.034
Long-term all-cause mortality^#^						
SHR (continuous)	511 (23.6)	1.30 (1.19–1.41)	< 0.001	632 (29.1)	1.26 (1.01–1.57)	0.038
SHR (categorical)						
Quartile 1	120 (22.1)	1.21 (0.92–1.57)	0.170	170 (31.3)	1.11 (0.88–1.41)	0.364
Quartile 2	103 (19.0)	Ref		133 (24.5)	Ref	
Quartile 3	110 (20.4)	1.23 (0.94–1.61)	0.140	145 (26.8)	1.04 (0.82–1.33)	0.735
Quartile 4	178 (32.8)	1.63 (1.27–2.09)	< 0.001	184 (33.9)	1.32 (1.05–1.66)	0.017

Both cohorts adjusted for age, gender, hypertension, diabetes mellitus, congestive heart failure, chronic kidney disease, atrial fibrillation, stroke, anemia, and revascularization;

#: Maximum follow-up of 12.1 years for American MIMIC-IV cohort, and 14.1 years for Chinese CIN-II cohort

Similar results were observed in Chinese CIN-II group. Each unit increase in the SHR index were associated with 34% increased risk of 1-year mortality (HR = 1.34 95%CI: 1.01–1.78, P = 0.043) and 26% increased risk of long-term mortality (HR = 1.26 95%CI: 1.01–1.57, P = 0.038). Patients with SHR ≥ 1.23 (quartile 4) also had a higher risk of 1-year (HR = 1.44 95%CI: 1.03–2.02, P = 0.034) and long-term (HR = 1.32 95%CI: 1.05–1.66, P = 0.017) all-cause mortality than quartile 2. (Table 2)

### Subgroup analyses

Subgroup analyses were performed for evaluation of the association of SHR with the all-cause mortality according to diabetes status (with or without) in two cohorts. (Figure. 3)

Among the patients without diabetes, SHR in quartile 4 was significantly associated with an increased risk of all-cause mortality on both MIMIC-IV cohort (HR = 2.03, 95%CI: 1.43–2.89) and CIN-II cohort (HR = 1.74, 95%CI: 1.26–2.41), compared with SHR in quartile 2. However, no significant difference was observed among diabetes patients in two cohorts. (MIMIC-IV: P for interaction = 0.101; CIN-II: P for interaction = 0.008). Similar results were observed in 1-year mortality.

## Discussion

To the best of our knowledge, this study is the first to examine the association between stress hyperglycemia ratio and long-term all-cause mortality in patients with critical AMI. To achieve this goal, we verify the consistency of the results among American and Chinese cohorts. Our findings demonstrated that (1) AMI patients after admission in the ICU from U.S. and China cohorts with elevated SHR level is associated with higher 1-year and long-term all-cause mortality. (2) elevated SHR is significantly associated with higher risk of all-cause mortality in the critical AMI patients without diabetes in both two cohorts. Most importantly, this study provides a simple and efficient biomarker for risk classification of stress hyperglycemia in AMI patients who admit in intensive care unit.

Stress hyperglycemia has been proven to be a strong predictor of a higher risk of mortality and morbidity risk in patients with AMI [22–25]. Previously, admission glucose was questioned as a marker for stress hyperglycemia, and high admission glucose value doesn’t necessarily indicate an acute glucose increase in response to AMI, particularly in diabetes patients with poor glycemic control [26]. Thus SHR, an index of relative glycemia, was introduced with the aim of gaining new insights into the relationship between hyperglycemia and patient outcomes by correcting glucose levels for HbA1c. Yang et al. reported a J-shaped correlation of SHR and long-term adverse outcomes in 5,562 patients with acute coronary syndrome (ACS), with quintile 3 as the reference, higher SHR (quintile 4 and 5) and lower SHR (quintile 1 and 2) had increased risks of 2-year MACCE, and the highest SHR (quintile 5) had the most increase in risk by 49% [12]. Wei et al. also observed a J-shaped relationship between stress hyperglycemia and mortality outcomes in patients with ACS undergoing PCI over a median follow-up period of 32.7 months [27]. In this study, the highest SHR quartile increased the risk of all-cause mortality by 118% and 33% among critically ill patients with AMI in the American MIMIC-IV and Chinese CIN-II cohorts, respectively.

In emergency or critically sick patients, the ideal glycemic control target is a source of great debate. Some of the previous studies found that stress hyperglycemia in acute AMI was more likely to be associated with worse outcomes in non-diabetic patients than in patients with known diabetes [28]. A recent observational study with 6,287 STEMI patients demonstrated that the highest SHR quartile was significantly associated with worse outcomes in non-diabetic, instead of diabetic patients, during a 5-year follow-up [29]. Similarly, in Chinese cohort, SHR was significantly associated with the long-term all-cause mortality among critical AMI patients without diabetes (P for interaction = 0.008). It indicated that SHR have a better potential on glucose management than admission blood glucose index among patients with critical myocardial infarction.

Stress hyperglycemia emphasizes the relative acute rise of glycemia in response to stress reaction or severe disease [30]. It has been identified as an independent risk factor for poor prognosis in patients with ACS or AMI, it causes myocardial infarctions to be larger, portending a higher risk of adverse cardiovascular events [16, 23]. We speculate that mild to moderate stress hyperglycemia may play a protective role in the acute phase by increasing cell survival factors and decreasing apoptosis to reduce infarct size and improve systolic function, especially for ischemia [15, 31]. However, the excessive SHR may trigger inflammation and oxidative stress, aggravates the endothelial dysfunction of critically ill AMI patients. and induces a pro-thrombotic state [14]. Meanwhile, stress hyperglycemia is always accompanied by oxidative stress and inflammatory responses, increasing endothelial dysfunction, thrombosis, and ischemia-reperfusion injury, result in the aggravated myocardial injury [17]. During myocardial infarction, stress hyperglycemia will promote a pro-thrombotic state and activate the neuroendocrine system, causing excessive release of catecholamines and cytokines, damaged the endothelial function of blood vessels [30, 32]. Moreover, researchers contend that stress hyperglycemia may lead the increasing production of reactive oxygen species, inducing cardiomyocyte apoptosis and cardiac dysfunction [33]. In addition, SHR were found to be significantly associated with the long-term all-cause mortality among critical AMI patients without diabetes. Since the blood glucose level is usually higher in patients with diabetes before ACS, the threshold glucose level associated with poor prognosis might be raised [30].

In the current study, we evaluated the predictive value of SHR for long-term all-cause mortality in patients with AMI admitted into ICU in the Chinese CIN-II cohort, and we verified the findings in the American MIMIC-IV cohort. We find higher SHR is associated with an increased risk of all-cause mortality among critically ill patients with AMI, and this relationship exists only among those who do not have diabetes. Patients admitted into ICUs tend to present with unstable hemodynamics and require optimal care and management in time. Our findings emphasize the importance of careful glycemic control in AMI patients admitted into ICUs in hope to improve patient outcomes. Moreover, patients with abnormally elevated SHR at admission should be treated with different glycemic management strategies, especially for those without diabetes. Further studies are needed determine the mechanisms between stress hyperglycemia and outcomes in critical AMI patients.

### Limitation

We acknowledge several limitations in our study. First, due to the nature of retrospective analysis, this study lacks data on potential confounders such as duration of diabetes. While it is the real-world study to demonstrate the significant relationship of elevated SHR on long-term all-cause mortality among AMI patients with critical status, and further describe the consistent on American and China cohorts. Second, patients with normal blood glucose or without history of diabetes were less likely to have HbA1c measurement. However, we could still observe the stratified relationship of mortality under different SHR levels on these two cohorts. Thirdly, both American MIMIC-IV and Chinese CIN-II cohorts are missing data about TIMI scores, a risk assessment tool used in patients with acute coronary syndromes. Therefore, as an alternative, we used the qSOFA score, which can promptly identify infected patients likely to fare poorly, as a risk assessment tool used in patients with acute coronary syndromes [34]. We have performed subgroup analysis according to qSOFA score (≥ 2 or < 2) and showed the results in Supplemental Table 1. Finally, this study only covered all-cause mortality, and did not include major adverse cardiovascular events. But this study was linked to survival information from Centers for Disease Control and Prevention, and have two large-scale critical AMI samples to evaluate the heterogeneity. Further perspective studies are warranted to evaluate the association of SHR and long-term prognosis among critical AMI patients.

## Conclusion

Our study shows that the SHR index shows a J-shaped association with 1-year and long-term all-cause mortality in both American MIMIC-IV and Chinese CIN-II cohort. SHR can be a prominent risk predictor of prognosis for AMI patients with critical status, especially in those without diabetes mellitus. Further randomized studies are needed to investigate the effect of glycemic control according to the SHR on improving outcomes among patients with critical AMI.

### Electronic supplementary material

Below is the link to the electronic supplementary material.

Supplementary Material 1

## Data Availability

The datasets used and/or analyzed during the current study are available from the corresponding author on reasonable request.
