# Supplementary material for: Impact of stress hyperglycemia ratio on mortality in patients with critical acute myocardial infarction: insight from american MIMIC-IV and the chinese CIN-II study
Source: Cardiovasc Diabetol. 2023 Oct 21;22:281. doi: 10.1186/s12933-023-02012-1 (PMC10589959; doi:10.1186/s12933-023-02012-1)
Supplement: Supplementary file 1 — Supplementary Material 1 [file 12933_2023_2012_MOESM1_ESM.docx]

**Supplemental table 1. The associations of SHR and all-cause mortality among patients with qSOFA score ≥2 or <2 in U.S. and Chinese critical AMI patients**

| SHR groups | MIMIC-IV | | | CIN-II | | |
| --- | --- | --- | --- | --- | --- | --- |
|  | HR (95%CI) | P value | P for interaction | HR (95%CI) | P value | P for interaction |
| 1-year all-cause mortality | | | | | | |
| qSOFA score ≥2 |  |  | 0.026 |  |  | 0.814 |
| Quartile 1 | 2.24 (0.9-5.58) | 0.084 |  | 0.84 (0.45-1.56) | 0.578 |  |
| Quartile 2 | Ref | - |  | Ref | - |  |
| Quartile 3 | 2.15 (0.85-5.43) | 0.104 |  | 0.65 (0.31-1.35) | 0.249 |  |
| Quartile 4 | 2.53 (1.07-5.97) | 0.035 |  | 1.12 (0.62-2.02) | 0.718 |  |
| qSOFA score <2 |  |  |  |  |  |  |
| Quartile 1 | 1.05 (0.74-1.50) | 0.785 |  | 1.50 (0.82-2.73) | 0.184 |  |
| Quartile 2 | Ref | - |  | Ref | - |  |
| Quartile 3 | 1.17 (0.83-1.65) | 0.363 |  | 1.33 (0.69-2.56) | 0.388 |  |
| Quartile 4 | 1.77 (1.30-2.41) | <0.001 |  | 1.29 (0.70-2.39) | 0.419 |  |
| Long-term all-cause mortality^#^ | | | | | | |
| qSOFA score ≥2 |  |  | 0.170 |  |  | 0.594 |
| Quartile 1 | 1.75 (0.77-4.00) | 0.181 |  | 0.89 (0.51-1.53) | 0.665 |  |
| Quartile 2 | Ref | - |  | Ref | - |  |
| Quartile 3 | 2.06 (0.89-4.75) | 0.091 |  | 0.60 (0.31-1.14) | 0.118 |  |
| Quartile 4 | 2.41 (1.10-5.30) | 0.028 |  | 1.00 (0.58-1.69) | 0.986 |  |
| qSOFA score <2 |  |  |  |  |  |  |
| Quartile 1 | 1.12 (0.84-1.50) | 0.424 |  | 1.34 (0.92-1.96) | 0.125 |  |
| Quartile 2 | Ref | - |  | Ref | - |  |
| Quartile 3 | 1.13 (0.84-1.51) | 0.414 |  | 1.15 (0.75-1.74) | 0.524 |  |
| Quartile 4 | 1.52 (1.16-1.98) | 0.002 |  | 1.39 (0.95-2.06) | 0.093 |  |

SHR = stress hyperglycemia ratio; qSOFA = quick sequential organ failure assessment

Both cohorts adjusted for age, gender, hypertension, diabetes mellitus, congestive heart failure, chronic kidney disease, atrial fibrillation, stroke, anemia, and revascularization;

#: Maximum follow-up of 12.1 years for American MIMIC-IV cohort, and 14.1 years for Chinese CIN-II cohort.
